# Electrochemical Immunosensor Using COOH-Functionalized 3D Graphene Electrodes for Sensitive Detection of Tau-441 Protein

**DOI:** 10.3390/bios15070465

**Published:** 2025-07-19

**Authors:** Sophia Nazir, Muhsin Dogan, Yinghui Wei, Genhua Pan

**Affiliations:** 1 Nanomaterials and Devices Laboratory (NMD), School of Engineering, Computing and Mathematics, University of Plymouth, Devon PL4 8AA, UK; 2 School of Engineering, Computing and Mathematics, University of Plymouth, Devon PL4 8AA, UK; 3 Biomedical Engineering, Engineering and Architecture Faculty, Izmir Bakircay University, 35665 Izmir, Turkey

**Keywords:** Alzheimer’s disease (AD), tau-441, carboxyl-modified, graphene foam electrode (GF), electrochemical immunosensor, biosensor

## Abstract

Early diagnosis of Alzheimer’s disease (AD) is essential for effective treatment; however current diagnostic methods are often complex, costly, and unsuitable for point-of-care testing. Graphene-based biosensors offer an alternative due to their affordability, versatility, and high conductivity. However, graphene’s conductivity can be compromised when its carbon lattice is oxidized to introduce functional groups for biomolecule immobilization. This study addresses this challenge by developing an electrochemical immunosensor using carboxyl-modified commercial graphene foam (COOH-GF) electrodes. The conductivity of graphene is preserved by enabling efficient COOH modification through π–π non-covalent interactions, while antibody immobilization is optimized via EDC-NHS carbodiimide chemistry. The immunosensor detects tau-441, an AD biomarker, using differential pulse voltammetry (DPV), achieving a detection range of 1 fM–1 nM, with a limit of detection (LOD) of 0.14 fM both in PBS and human serum. It demonstrates high selectivity against other AD-related proteins, including tau-217, tau-181, amyloid beta (Aβ_1-40_ and Aβ_1-42_), and 1% BSA. These findings underscore its potential as a highly sensitive, cost-effective tool for early AD diagnosis.

## 1. Introduction

Alzheimer’s disease (AD) is a severe neurodegenerative disorder characterized by progressive cognitive decline and functional impairment, ultimately leading to death [1]. It is the most common form of dementia, affecting millions of elderly individuals worldwide [2]. It is characterized by the formation of amyloid-beta (Aβ) plaques and neurofibrillary tangles made up of hyperphosphorylated tau proteins. Tau is a microtubule-associated protein normally found in axons, where it stabilizes the cytoskeletal structure. Under pathological conditions, tau becomes abnormally phosphorylated, leading to neuronal dysfunction and degeneration. In AD patients, total tau and phosphorylated tau proteins have emerged as critical biomarkers for diagnosis and disease monitoring due to their strong association with disease progression [3,4]. Current methods for evaluating phosphorylation of tau include enzyme-linked immunosorbent assays (ELISA) [5], polymerase chain reaction (PCR) [6], fluorescent immunosensors [7], surface plasmon resonance (SPR) [8,9], and piezoelectric biosensors [10]. However, these conventional techniques often lack the sensitivity needed to detect phosphorylated tau at early stages of disease. For example, while ELISA is effective for detecting tau proteins, it is insufficiently sensitive for identifying early-stage disease, resulting in diagnostic blind spots during critical windows. Furthermore, tau phosphorylation is not unique to AD disease [11], it also occurs in other neurodegenerative disorders, reducing its diagnostic specificity as a diagnostic marker. These limitations underscore the need for advanced technologies capable of detecting AD with high sensitivity and specificity.

To date, various advanced methods have been developed for tau protein detection, including chemiluminescence ELISA [12], electrochemiluminescence [13], and nanopores [14]. However, these techniques face drawbacks such as complex instrument maintenance, unstable luminescent reagents, and susceptibility to interference. Electrochemical immunosensing technology, distinguished by its rapid response, high sensitivity, and miniaturization potential, has emerged as a superior alternative [15,16]. Novel electrochemical detection methods based on tau are paving the way for precise monitoring. For instance, Arjun et al. [17] developed a novel vanadium MXene-based sensor for the highly sensitive detection of tau proteins. The analytical performance of electrochemical sensors heavily depends on the functional properties of electrode substrate modification materials. Thus, developing highly conductive and high-performance functional materials is central to optimizing sensor design and enhancing performance [18,19].

Metal–organic frameworks (MOFs), characterized by their high surface area, abundant active sites, and tuneable functionality, exhibit unique advantages in catalysis, adsorption, and sensing [20]. Yuan et al. [21] reported a Fe-modified MOF bidirectional regulator for electrochemiluminescence with superior sensing performance, constructing an enzyme-free electrochemical sensing of tau detection. The ECL sensor showed good analytical performance with a detection limit as low as 3.38 fg mL^−1^. Gold nanoparticles (AuNPs), renowned for their high conductivity and biocompatibility, are widely used in electrochemical interface modification. Natale et al. [22] fabricated a sensitive colorimetric immunosensor using AuNP-functionalized polymer film for specific detection of tau proteins, a biomarker for AD detection. Building on the complementary advantages of AuNPs and polymer film, this study proposes embedding AuNP-functionalized polymer film or surfaces to enhance overall conductivity and charge transfer efficiency. Khiavi et al. [23] developed a sensor with a detection limit as low as 1 fg mL^−1^ for cis-p-tau, validating the efficacy of this composite strategy in electrochemical detection in the serum of AD patients, indicating its accuracy and feasibility for real-sample analysis.

Electrochemical biosensors rely on materials with a high surface-area-to-volume ratio such as conductive or semiconductive compounds to increase the number of anchoring sites and boost sensitivity. Research has shown that carbon nanomaterials are particularly effective for developing flexible, stretchable transducers suited to biological applications [24]. Graphene is a widely investigated material due to its better conductivity than carbon nanotubes [25,26]. It can function as an active channel, dielectric, or conductive electrode. However, to enable biomolecule attachment, graphene often requires modification, through oxidation of the graphitic lattice. This process introduces defects primarily at the edges of the nanostructures, and these defects reduce their overall electronic conductivity. Additionally, covalent surface modifications may introduce defects and modify graphene electrode properties [27,28]. Consequently, non-covalent modification strategies are preferred, for example, a commercially available carboxyl-modified three-dimensional (3D) graphene foam (COOH-GF) electrode. Biochar-based screen-printed electrodes can indeed serve as low-cost sustainable platforms for antibody immobilization without requiring sophisticated materials [29,30]. While biochar offers sustainability and native carboxyl groups, its use in electrochemical biosensors is limited by poor electrical conductivity, structural heterogeneity, and lower reproducibility [31] compared to COOH-functionalized 3D graphene foam (COOH-GF). Whether employed as a carrier, a catalyst, or an absorbent, the potential interference from the initial biomass (such as heavy metals) must be taken into account. This is because such interference could contaminate the analytes or impact the sensors’ repeatability, which is a crucial technical metric for assessing sensors and biosensors [32,33].

Graphene-foam-based electrodes, a 3D porous structure of graphene, offer unique advantages including a large surface area, excellent conductivity, structural stability, and biocompatibility. Its porous architecture facilitates efficient biomolecule loading while enhancing electron transport and antibody–antigen interactions [18,34]. Functionalization with carboxyl groups further improves biosensor performance by introducing active sites for covalent biomolecule attachment, thereby enhancing antibody immobilization efficiency while providing a robust platform for signal generation [35].

In this study, we present a simple and ultrasensitive electrochemical immunosensor employing a commercially available carboxyl-modified graphene foam (COOH-GF) electrode as the sensing substrate for tau-441 protein detection. Antibodies were immobilized by activating the π-π-linked carboxyl functional groups using EDC-NHS chemistry. The electrical properties of graphene enabled direct electron transfer from the capacitive double layer to the Gii-sense graphene foam transducing layer after biocontact. Electrochemical measurements were performed in phosphate-buffered saline (PBS, pH 7.4), which is a suitable medium for use in both home and clinical settings. In addition, human serum was used to validate the applicability of the biosensor in real-sample analysis. Furthermore, our method decreased handling while achieving detection limits in the femtomolar range through uncomplicated measurement. The integration of carboxyl-modified graphene foam (COOH-GF) into electrochemical sensors paves the way for the development of cost-effective diagnostic tools with broad applications in clinical research.

## 2. Materials and Methods

### 2.1. Chemicals

The COOH-modified Gii-sense graphene foam (GF) electrodes were purchased from Integrated Graphene Ltd. (Stirling, UK) (Figure S1). Sigma Aldrich (Gillingham, UK) supplied the following: bovine serum albumin (BSA), potassium ferricyanide (K_3_Fe(CN)_6_), potassium chloride (KCl), phosphate-buffered saline (PBS), N-(3-Dimethylamino-propyl)-N′-ethyl carbodiimide hydrochloride (EDC), N-Hydroxysuccinimide (NHS), Human Aβ_1-42_ peptides, human serum (H3667), and Eppendorf^®^ LoBind microcentrifuge tubes (Gillingham, UK). PBS tablets, pH 7.4, were purchased from Fisher Scientific (Loughborough, UK), and the PBS buffer solution was prepared in Milli-Q water. Human Aβ_1-40_ peptides were obtained from Tocris Bioscience (Abingdon, UK). Anti-tau-441 monoclonal antibody (mAb) and tau-441 peptide, tau-217 peptides, and tau-181 peptides were obtained from Abcam (Cambridge, UK). All reagents were used exactly as described, unless otherwise noted. Deionized water (18.2 MΩ·cm at 25 °C) from an ELGA water purification system was used for preparing standard solutions and buffers.

### 2.2. Instruments and Characterization

SEM analyses were obtained using a scanning electron microscope (LV-SEM, Jeol, 6610, Tokyo, Japan), providing a high-resolution image. A μstat ECL potentiostat was obtained from Metrohm Dropsens (Runcorn, UK), and DropView 8400 2.0 software was used to manage it during cyclic voltammetry (CV) and differential pulse voltammetry (DPV) measurements.

### 2.3. Preparation of the Tau Biosensor

**Figure 1:**
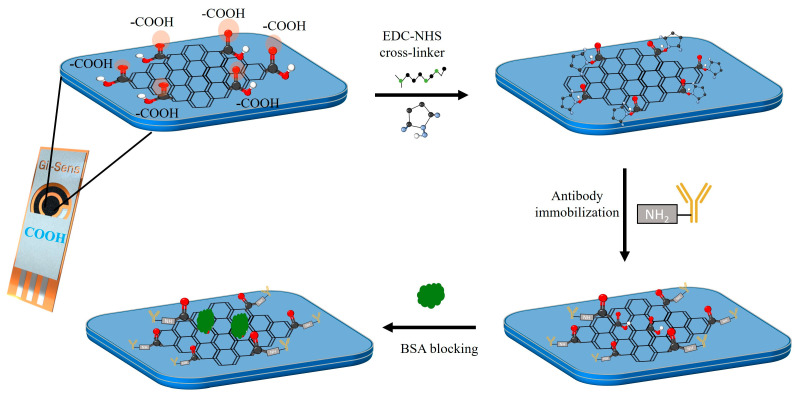
Schematic representation of the immobilization step for the tau antibody onto COOH-modified graphene electrodes (COOH-GF) via EDC-NHS coupling.

A 0.1 M NHS solution and a 0.4 M EDC solution were prepared in PBS (pH 7.4) and stirred until homogeneous. The solutions were mixed in a 1:1 ratio, and the COOH-GF-based electrodes were immersed in the resulting solution for 1 hour to activate the -COOH group. EDC acts as a coupling agent, converting the -COOH group into an amine-reactive ester to bind to the -NH_2_ of the tau-441 antibody, as shown in Figure S2 [36]. The crosslinked electrode was rinsed with PBS (pH 7.4) and dried under nitrogen. A 20 µL aliquot of a 20 µg/ml anti-tau-441 monoclonal antibody (mAb) solution prepared in PBS (pH 7.4) was applied to the working electrode. After 2 hours of incubation at room temperature (RT), the electrode was rinsed with PBS (pH 7.4) and deionized water (DI), and non-specific sites were blocked by incubating with 20 µL of 0.1% BSA in PBS (pH 7.4) buffer for 30 minutes at RT. After that, it was washed with PBS (pH 7.4) and dried under nitrogen. All incubation times were optimized for maximum sensitivity of the biosensor. Schematic of the functionalization steps involved in the preparation of biosensors are shown in Figure 1.

### 2.4. Electrochemical Measurements

For the electrochemical experiments, a 10 mM K_3_[Fe(CN)_6_] and a 1 M KCl K3[Fe(CN)6] solution served as the supporting electrolyte [37]. CV measurements were performed at a scan rate of 50 mV/s, ranging from 0.3 to −0.4 V. DPV scans were measured over a potential range of between 0.30 V and −0.45 V, using the following parameters: scan rate of 100 mV/s and pulse time of 0.4 s.

### 2.5. Determination of Tau-441

Tau-441 peptides in various concentrations were prepared in PBS (pH 7.4) and human serum by vortex mixing for 20 s. Throughout the experiments, all the concentrations were stored on ice. After applying 20 µL of each peptide solution to the biosensor, it was allowed to incubate at RT for 1 h. To remove any remaining peptides, the sensor was cleaned once with PBS (pH 7.4). Each sensor measurement took roughly three to four minutes.

## 3. Results

### 3.1. Surface Characterization

**Figure 2:**
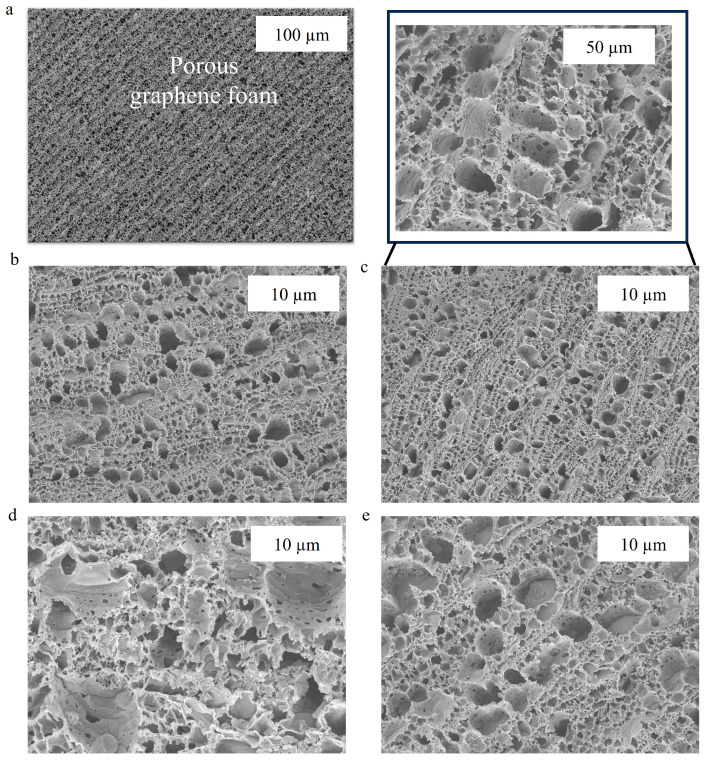
SEM images of (**a**) bare GF electrodes, (**b**) COOH-GF, (**c**) EDC-NHS activation of COOH-GF, (**d**) mAb modified COOH-GF, (**e**) and BSA-blocked COOH-GF electrodes at 10,000 × magnifications.

Scanning electron microscopy (SEM) was employed to examine the topographical features of working electrodes at various stages of functionalization, including carboxyl (-COOH) group addition, activation with EDC-NHS, tau antibody immobilization, and BSA blocking. These SEM observations suggest that the biosensing interface was successfully fabricated sequentially. Figure 2a demonstrates that bare 3D graphene foam, with its large surface area and three-dimensional scaffold, has a highly porous and interconnected network architecture. This architecture optimizes electron transport during biosensing and can handle high biomolecule loads. No notable structural changes were seen at the macroscale after the insertion of carboxyl functional groups; however, there was a modest increase in surface texture on the foam walls, indicating that the graphene surface had been chemically modified (Figure 2b). The carboxyl groups were then activated with EDC and NHS, resulting in a more textured surface at the nanoscale, which is consistent with the production of NHS ester intermediates ready for covalent coupling (see Figure 2c). This procedure prepares the foam for maximum antibody adherence. SEM images after mAb immobilization demonstrated a distinct granular or clustered deposition along the foam walls’ interior surfaces, as shown in Figure 2d. Finally, the bovine serum albumin (BSA) blocking process resulted in a smoother coating over the interior surfaces, indicating uniform coverage that lowers nonspecific binding and boosts biosensor selectivity (Figure 2e).

Overall, by monitoring alterations in surface texture and the distribution of living molecules within the 3D graphene foam, SEM imaging successfully validates each functionalization stage. Better biomolecule immobilization is made possible by the foam’s large surface area, which instantly improves the biosensor’s performance and sensitivity.

### 3.2. Tau Biosensor Construction

The EDC-NHS coupling chemistry was used for the activation of carboxyl-modified graphene electrodes (COOH-GF). This process activates the carboxyl groups on the GF electrodes, enabling covalent attachment of tau-441 antibodies. The electrical π-clouds of graphene enhance the activity of these carboxyl groups, facilitating efficient biomolecule attachment and signal transmission. The π-π interactions between graphene and aromatic carboxylic acid groups allow for non-covalent functionalization, which preserves graphene’s electrical structure while giving stable surface modification. These interactions improve electron transfer by increasing π-orbital overlap, lowering the energy barrier for charge transport. As a result, they help to improve signal stability and amplification in electrochemical and biosensing systems. These interactions are commonly used in functional nanomaterials and sensor platforms [38,39]. Before starting the experiments, six different electrodes were used to measure the DPV signal in the experimental setup to examine the repeatability of the GF electrodes (Figure S3). The relative standard deviation demonstrates excellent reproducibility and confirms this electrode’s accuracy. This demonstrates the reliability and stability of COOH-GF electrodes during fabrication and electrochemical testing, critical factors for consistent biosensor performance.

Electrical characterization was performed before and after the activation of carboxyl groups with EDC-NHS on COOH-GF electrodes (Figure S4). The results showed improved electrochemical performance following activation, attributed to enhanced biomolecule immobilization and increased active sites. This functionalization is crucial for biosensor sensitivity, though it does not directly enhance conductivity. Further testing was conducted on tau COOH-GF biosensors in PBS (pH 7.4) buffer (Figure S5a). When varying scan rates from 0.01 V to 0.1 V over a potential range from 0.2 V to −0.4 V using a 10 mM K_3_[Fe(CN)_6_] and a 1 M KCl (Figure S5b), a linear trend was observed (R^2^ = 0.99). This indicates the redox process is predominantly diffusion-controlled under these experimental conditions.

### 3.3. Characterization of the Working Electrodes

**Figure 3:**
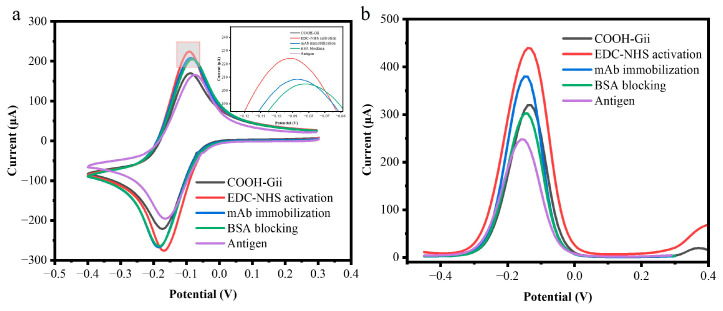
(**a**) Cyclic voltammograms depicting each surface modification measurements of a 10 mM [Fe(CN)_6_]^3−^ and 1M KCl with COOH-GF electrodes (black), activation with EDC-NHS/COOH-GF electrodes (red), mAb modified COOH-GF (blue), BSA blocking COOH-GF electrodes (green), and antigen binding (purple). (**b**) differential pulse voltammetry (DPV) measurements with a 10 mM K_3_[Fe(CN)_6_] and a 1 M KCl solution with COOH-GF electrodes (black), activation with EDC-NHS/COOH-GF electrodes (red), mAb modified COOH-GF (blue), BSA blocking COOH-GF electrodes (green), and antigen binding (purple).

The electrochemical characterization of the working electrode surfaces is depicted in Figure 3. Following each stage of assembly, CV was used for the electrochemical characterization of the electrode surfaces. In the potential range of −0.45 V to +4.0 V, all measurements were carried out at a scan rate of 50 mV/s in a 1 mM KCl solution containing 10 mM of the redox probe 10 mM K_3_[Fe(CN)_6_]. All electrodes showed distinctive anodic and cathodic peaks, as shown in the voltammograms. When using bare COOH-GF electrodes, the peak current was around 170 µA (black peak). Following EDC/NHS activation, the cyclic voltammogram’s faradic currents rise, whereas the red curve shows a decrease in peak separation (ΔEp). This could be because of their EDC-NHS interaction, which decreases the number of free carboxyl groups on the working electrode.

This depletion results in a decrease in the repulsive force across the free carboxyl groups and the [Fe(CN)_6_]^3−^ anions. The peak current (blue curve) decreases when m-Ab is covalently immobilized onto the active surface of a COOH-GF electrode. This is because the immobilized antibodies act as a non-conductive organic barrier that blocks electron transfer and prevents the redox probe [Fe(CN)_6_]^3−/4−^ from diffusing to the electrode surface. Finally, blocking unreacted carboxylic groups with bovine serum albumin (BSA) further reduced the current response to 204 µA (green curve), confirming successful surface modification at each step. A similar trend was observed in negative peak currents after each modification. These findings show that antibodies were successfully immobilized at the electrode surface. Following target recognition, the peak current decreased because the tau-441 antigen and the monoclonal antibody’s specific binding served as a kinetic barrier on the electrode surface, preventing redox particles from penetrating the layers and reaching the conductive electrode surfaces.

DPV was also performed to monitor biomolecule immobilization on the electrode surface, as shown in Figure 3b. The DPV results followed a similar trend to CV measurements. The peak current decreased from 287 µA for the COOH-GF electrode to 225 µA for the fully modified antigen/BSA/anti-mAb/EDC-NHS/COOH-GF electrode (purple). The DPV peak current decreased progressively after each modification step, related to the tau-441 antigen, due to the hindered diffusion of the redox label to the electrode surface, derived from both the carboxyl groups and the attached proteins and biological molecules.

### 3.4. Optimization of the Experimental Conditions

We optimized several experimental parameters that might impact the immunosensor’s sensitivity and detection limit. The quantity of biomolecules immobilized on the electrode was strongly affected by the concentrations of the antibody and EDC-NHS cross-linker, as well as the incubation duration. Figure S6a illustrates that the activation time of the COOH-modified GF electrode using EDC-NHS did not significantly affect the current; once the incubation time exceeded 1 hour, the current remained relatively unchanged. Additionally, the number of immobilized biomolecules was dependent on both the length of the incubation period and the antibody concentration, as shown in Figure S6b.

After more than 2 hours of incubation, as depicted in Figure S6c, the antibody concentration exceeded 20 μg/mL, and the current approached a plateau. To optimize the antigen incubation duration, working electrodes were incubated with tau-441 for varying lengths of time (15, 30, 45, 60, and 75 min). Figure S6d shows that the change in current (Δ*I*) stabilized after 1 hour, indicating that the antigen–antibody immunoreaction had been completed. Therefore, we selected 1 h as the ideal incubation time for further analyses, maintaining pH 7.4 for optimal performance.

### 3.5. Analytical Performance of the Tau Biosensor

**Figure 4:**
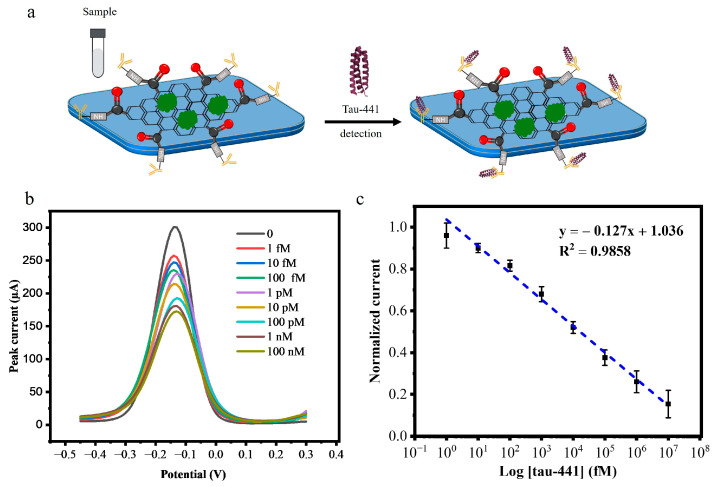
(**a**) Addition of the sample to the tau biosensor. (**b**) DPV response curve of BSA/mAb/EDC-NHS/COOH-GF modified electrode incubated with concentrations of tau-441 (1 fM, 10 fM, 100 fM, 1 pM,10 pM, 100 pM, 1 nM, 10 nM, and 100 nM). (**c**) Calibration curve for the change in current corresponding to the tau-441 concentration for the tau biosensor (*n* = 3).

After optimizing the experimental parameters, the performance of the biosensor was assessed using DPV against 20 μL of antigen (tau-441 peptide) at the following concentrations: 1 fM, 10 fM, 100 fM, 1 pM, 10 pM, 100 pM, 1 nM, 10 nM, and 100 nM. Following a 1-hour incubation, as shown in Figure 4a, 75 μL of supporting solution was added, and the DPV signal was monitored. After each incubation with a given concentration of tau-441, the sensor was rinsed thoroughly with PBS (pH 7.4) and DI water to remove unbound protein. As the concentration of the antigen (tau-441 peptides) increased, the peak current decreased because the protein binds to the tau antibody, preventing electron transfer as depicted in Figure 4b. Figure 4c shows the calibration curve, plotting the log of concentration (in fM) versus normalized current. The calibration curve exhibited a linear relationship between the analyte concentration and the normalized current response described equation y = −0.127x + 1.036 with a high coefficient (R^2^ = 0.98). The negative slope indicates that increasing analyte concentration progressively decreases the current signal due to the formation of an insulating layer on the electrode surface that impedes electron transfer. The normalized current decreased linearly (R^2^ = 0.98) as the biomarker concentration increased (*n* =3).

After establishing the calibration curve, we calculated the limit of detection (LOD) for our tau biosensor. The LOD was calculated following the formula: LOD = 3.3 σ/S. Here, σ is the standard deviation of the blank measurements, and S is the slope of the calibration curve in the linear range.

This exceptionally low LOD of 0.14 fM demonstrates the ultrasensitive detection capability of our tau biosensor. The high sensitivity can be attributed to the large surface area of the graphene foam electrode, which allows for a greater number of immobilized antibodies and the excellent conductivity of the COOH-modified GF electrodes, which allows for fast electron transport.

### 3.6. Stability and Selectivity of the Tau Biosensor

**Figure 5:**
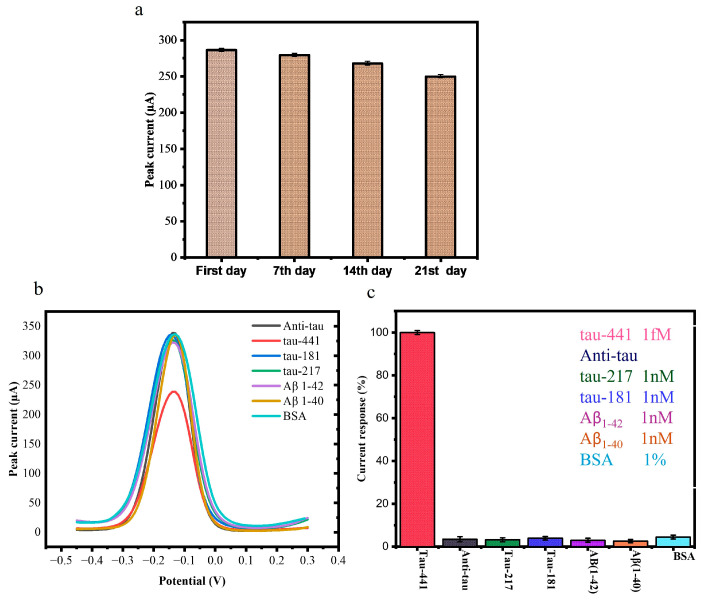
(**a**) Results of DPV responses of BSA/mAb/EDC-NHS/COOH-GF into 10 mM K_3_[Fe(CN)_6_] and 1 M KCl and 100 fM tau-441 at a scanning rate of 100 mV/s on the first day and after storage for one, two, and three weeks at 4 °C. (**b**) DPV profile of interference effect of tau-217, tau-181, Aβ_1-40_, Aβ_1-42_, and 1% BSA. (**c**) Interference effect of tau-217, tau-181, Aβ_1-40_ and Aβ_1-42_, and 1% BSA on BSA/anti-mAb/EDC-NHS/COOH-GF modified electrodes.

Following three weeks of storage at 4 °C, the stability of the BSA/mAb/EDC-NHS/COOH-GF electrodes was assessed. Figure 5a displays the results of the DPV responses of BSA/Anti-mAb/EDC-NHS/COOH-GF electrodes in a solution containing [Fe(CN)_6_]^3−^ 10 mM K_3_[Fe(CN)_6_] and a 1 M KCl while 100 fM of tau-441 peptides at a scanning rate of 100 mV/s on the first day and after storage on the seventh, fourteenth, and twenty-first days at 4 °C. The results show that after three weeks, the electrochemical signal had decreased by less than 10%, suggesting that the suggested immunosensor is fairly stable.

One of the most important aspects of assessing the efficiency of the developed immunosensor was its specificity. To assess this, we tested the biosensor against (tau-217, tau-181) and amyloid beta peptide Aβ_1-40_ and Aβ_1-42_, which are commonly identified as AD biomarkers. We also measure 1% BSA to check the interference effect of proteins. We used a non-target concentration more than 20 times greater than that of target tau-441 (1 fM) to incubate the immunosensor. There were no significant changes or alterations in the DPV signal for these interferents (Figure 5b,c), confirming that the immunosensor could accurately detect the specific antigen.

The specificity studies were also conducted for each AD biomarker, which showed results of 3.251% for tau-217, 3.80% for tau-181, 3.0% for Aβ_1-42_, 2.60% for Aβ_1-42,_ and 4.21% for BSA. The obtained results were calculated as relative. This high specificity is due to the unique structure of the antibody, which ensures that the detection results are unaffected by other biomarkers. This demonstrates that our immunosensor exhibits strong selectivity and specificity for tau-441 [18,34].

### 3.7. Human Serum Analysis

**Figure 6:**
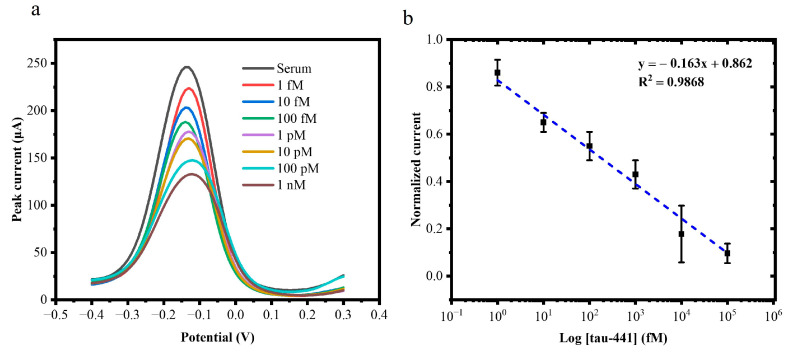
(**a**) DPV response of the tau biosensor to the increasing concentrations of target tau-441 peptides from 1 fM, 10 fM, 100 fM, 1 pM, 10 pM, 100 pM, and 1 nM. (**b**) Calibration curve for the change in current corresponding to the tau-441 concentration for the tau biosensor (*n* = 3).

To demonstrate the applicability of the developed tau biosensor in real biological samples, the biosensor was validated using human serum. Different concentrations of tau-441 peptides were prepared in human serum, i.e., 1 fM, 10 fM, 100 fM, 1 pM,10 pM, 100 pM, and 1 nM. The DPV curves at varying concentrations of human serum samples and their calibration plot are shown in Figure 6a,b. The sensing platform displayed high linearity in human serum with a correlation coefficient of R^2^ = 0.98. At *n* = 3, the limit of detection (LOD) was calculated to be approximately 1 fM for human serum samples. The obtained LOD was comparable to the electrochemical sensors that have been developed previously for tau protein (Table 1). These observations evidenced high sensitivity of the immunosensor for measurement of tau-441, either in PBS or a complex matrix such as serum.

## 4. Discussion

Previously, our group employed reduced graphene oxide (rGO) screen-printed electrodes to detect different amyloid beta proteins [45]. For instance, amine-functionalized (rGO) for Aβ_1-40_ and Aβ_1-42_ with an LoD of 9.51 fM and 8.65 fM [46]. However, these advanced materials and manufacturing procedures may make it challenging to reproduce the devices. In contrast, the tau biosensor developed in this study offers excellent sensitivity and accuracy. Its high accuracy stems from the combination of 3D graphene foam electrical characteristics and their electrical conductivity preservation after non-covalent modification with the COOH groups. The highly conductive Gii-sense electrode, modified with a COOH group, was used for this purpose due to its superior electron transport capabilities and ability to immobilize functional groups without requiring oxidation. The use of GF in its graphene foam form also prevents graphene flakes from restacking, thereby maintaining excellent conductivity. As a results of these features, the COOH-GF-based tau biosensor exhibited a broad linear detection range of 1 fM to 10 nM, with an outstanding limit of detection of 0.14 fM.

This study introduces a COOH-modified graphene foam electrochemical sensor as an effective tool for the sensitive and selective detection of tau proteins in PBS and human serum samples. The preliminary results indicate the successful development of this promising sensor for the early monitoring of tau protein levels, demonstrating excellent accuracy and stability.

Limitations: The activation of the COOH group on GF electrodes using EDC-NHS chemistry results in pyrene NHS esters that form a strong amine bond with the antibody [18], resulting in a target-specific platform. However, the biosensor fabrication process requires precise optimization of activation and incubation steps, particularly for effective antibody immobilization. A significant challenge lies in achieving uniform COOH group functionalization across the electrode surface, as the reaction may not proceed homogeneously [47]. Furthermore, it can be challenging to regulate the antibody orientation on the surface, which can lower the capture effectiveness. Future work is planned to overcome this challenge and work with clinical samples of blood and serum. Then, the performance will be validated against the gold-standard GC-MS method, underscoring its potential for point-of-care applications in metabolic health management.

## 5. Conclusions

In summary, a highly sensitive tau biosensor constructed on COOH-modified Gii-sense graphene foam electrodes detects tau-441 peptide. The biosensor has a high detection limit (0.14 fM) and a linear dynamic range (1 fM to 1 nM). This limit of detection is explained by the non-covalent modification of graphene electrodes with COOH, which prevents oxidation and preserves their intrinsic properties. The tau biosensor responds well due to its huge surface area and quick electron transport from 3D graphene. Additionally, the biosensor exhibits long-term stability and sensitivity in human serum samples. This study shows that the COOH/GF-modified sensor has significant promise for medical use and use at home in the rapid detection of AD biomarkers. The three-dimensional graphene-based biosensor fabrication process described in this study is easily extensible to the development of various biosensors based on other recognition components such as aptamers, antibodies, and nanobodies. Additionally, exploring its use in multiplexed detection systems could further enhance its utility in monitoring multiple biomarkers simultaneously.

## Figures and Tables

**Table 1:** Summary of various electrochemical biosensors for tau peptide measurements.

Type of Tau Peptide	Receptor	Method	Electrode Material	Linear Range	Matrix Type	Detection Limit	Ref.
tau-441	Antibody	DPV	GO/PBNCs/C-SPE	1.09–2.18 pmol/L	PBS	0.01 pmol/L	[40]
tau-441	PANI-Tau-441	DPV	Vanadium MXene polydopamine (Vx PDA)	122 aM/L to 122 pM/L	Interstitial fluid (ISF) and plasma	60 aM/L	[17]
tau-381	Antibody+aptamer	DPV, CV, and EIS	Gold nanoparticles (AuNPs)	0.5 pM–100 pM	Human serum	0.42 pM	[41]
tau-181	Aptamer	DPV, CV, and EIS	Glassy-carbon electrode	1 pM–100 pM	Human serum	0.70 pM	[42]
*cis* P-tau	DNAzyme	DPV, CV, and EIS	Gold electrode	10 × 10^−14^ M–3.0 × 10^−9^ M	PBS and serum	0.02–0.05 pM	[43]
tau-441	Antibody	SWV and CV	Reduced graphene oxide (rGO)	0.08–80 pM	Human serum	75 fM	[44]
tau-441	Antibody	DPV	COOH-GF	1 fM–1 nM	Human serum	0.14 fM	Present work

## Data Availability

The original contributions presented in this study are included in the article. Further inquiries can be directed to the corresponding author.

## Supplementary Materials

The following supporting information can be downloaded at: https://www.mdpi.com/article/10.3390/bios15070465/s1, Figure S1. COOH modified Gii-sense electrodes. Figure S2. Activation of COOH-GF electrodes with EDC-NHS carbodiimide chemistry. Figure S3. DPV signal measurement in the experimental setup in order to examine the repeatability of GF electrodes. Figure S4. Compare the electrical characteristics of the electrode before and after the activation of the carboxyl group. Figure S5. (a) Scan rate characterization from 0.01 V to 0.1 V by cyclic voltammetry characterization in a solution containing 10 mM K_3_[Fe(CN)_6_] and 1 M KCl onto the BSA/mAb/EDC-NHS/COOH-GF biosensor. (b) linear curve of anodic peak current. Figure S6. For the optimization of the experimental conditions, the DPV measurements were carried out in a solution containing 10 mM K_3_[Fe(CN)_6_] and 1 M KCl using COOH-GF electrodes to investigate the effects of: (a) activation time of the COOH groups with the EDC-NHS cross-linker, (b) the concentration of tau-441 antibody, (c) the antibody immobilization time, (d) the antigen (tau-441 peptide) immobilization time.
