# Supplementary material for: Electrochemical Immunosensor Using COOH-Functionalized 3D Graphene Electrodes for Sensitive Detection of Tau-441 Protein
Source: Biosensors (Basel). 2025 Jul 19;15(7):465. doi: 10.3390/bios15070465 (PMC12293263; doi:10.3390/bios15070465)
Supplement: Supplementary file 1 [file biosensors-15-00465-s001.zip › biosensors-3698676-supplementary.pdf]

Supplementary Information

Electrochemical Immunosensor using COOH-Functionalized 3D Graphene Electrodes for Sensitive Detection of Tau-441 Protein

Sophia Nazir [1,2]*, Muhsin Dogan [1,3] , Yinghui Wei[2] and Genhua Pan [1]

[1] Nanomaterials and Devices Laboratory, School of Engineering, Computing and Mathematics, University of Plymouth, Devon, PL4 8AA, UK
2 School of Engineering, Computing and Mathematics, University of Plymouth, Devon, PL4 8AA, UK
3 Biomedical Engineering, Engineering and Architecture Faculty, Izmir Bakircay University, 35665 Izmir, Turkey

* Correspondence: sophia.nazir@plymouth.ac.uk. sophia.nazir@gmail.com

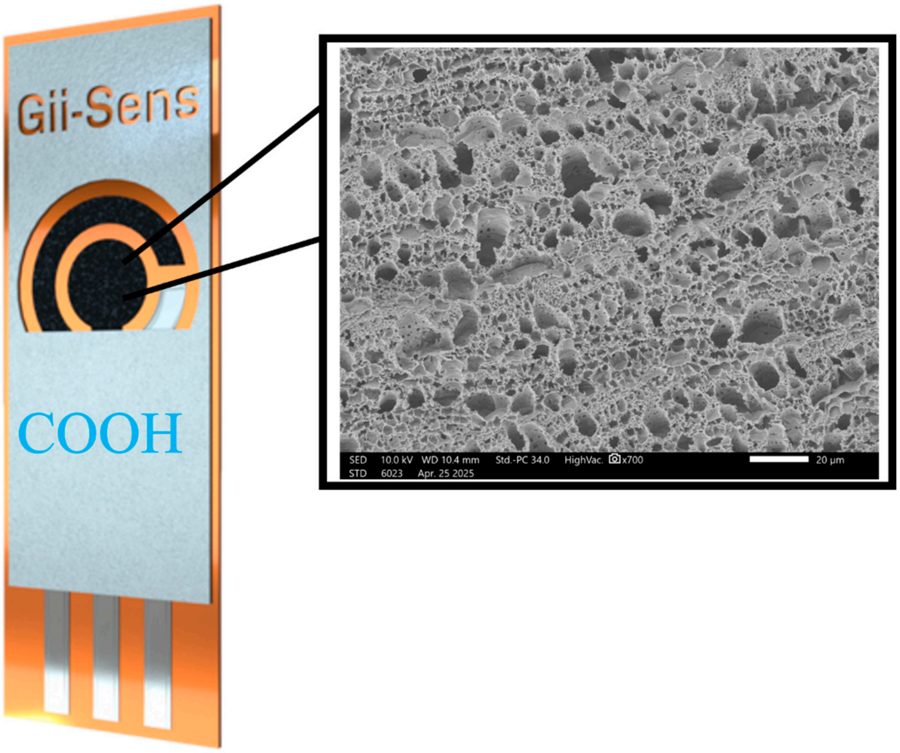

Figure S1. COOH modified Gii-sense electrodes.

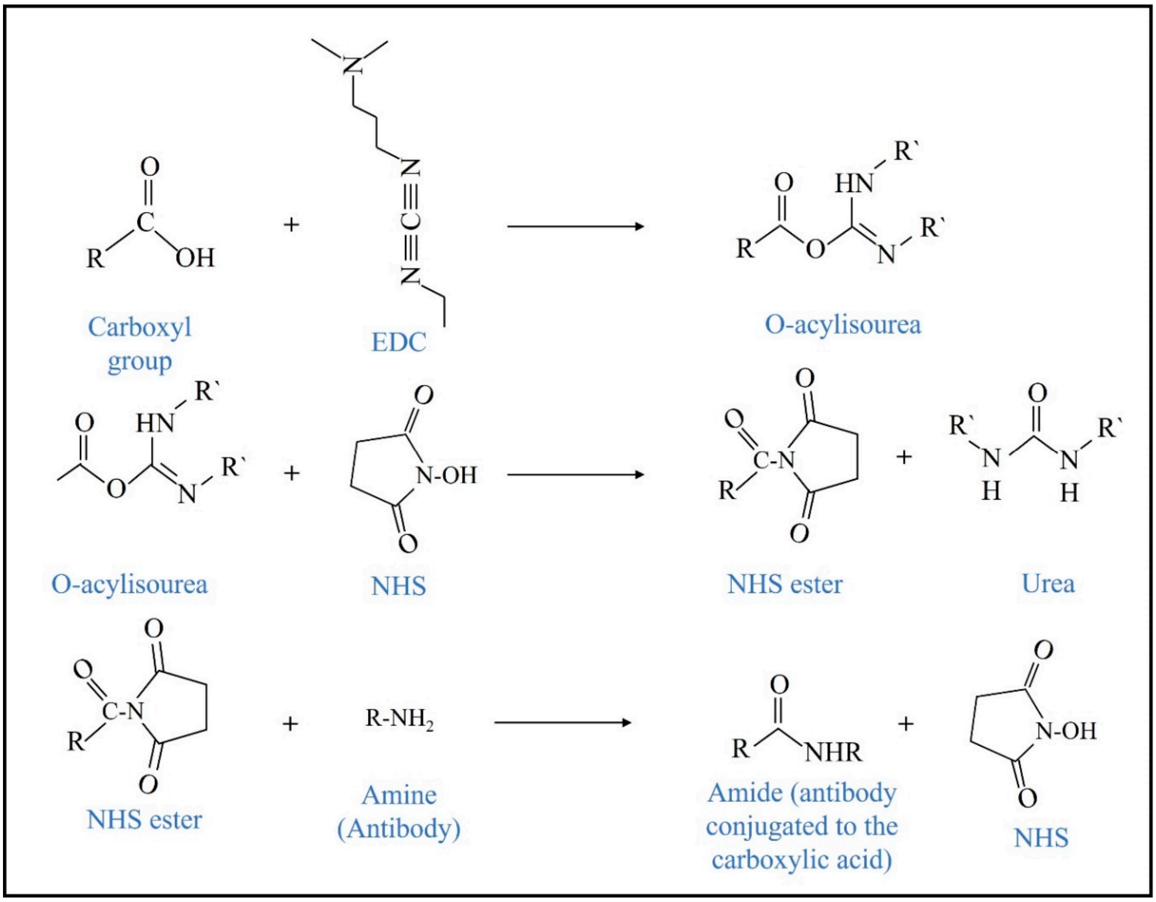

Figure S2. Activation of COOH-GF electrodes with EDC-NHS carbodiimide chemistry.

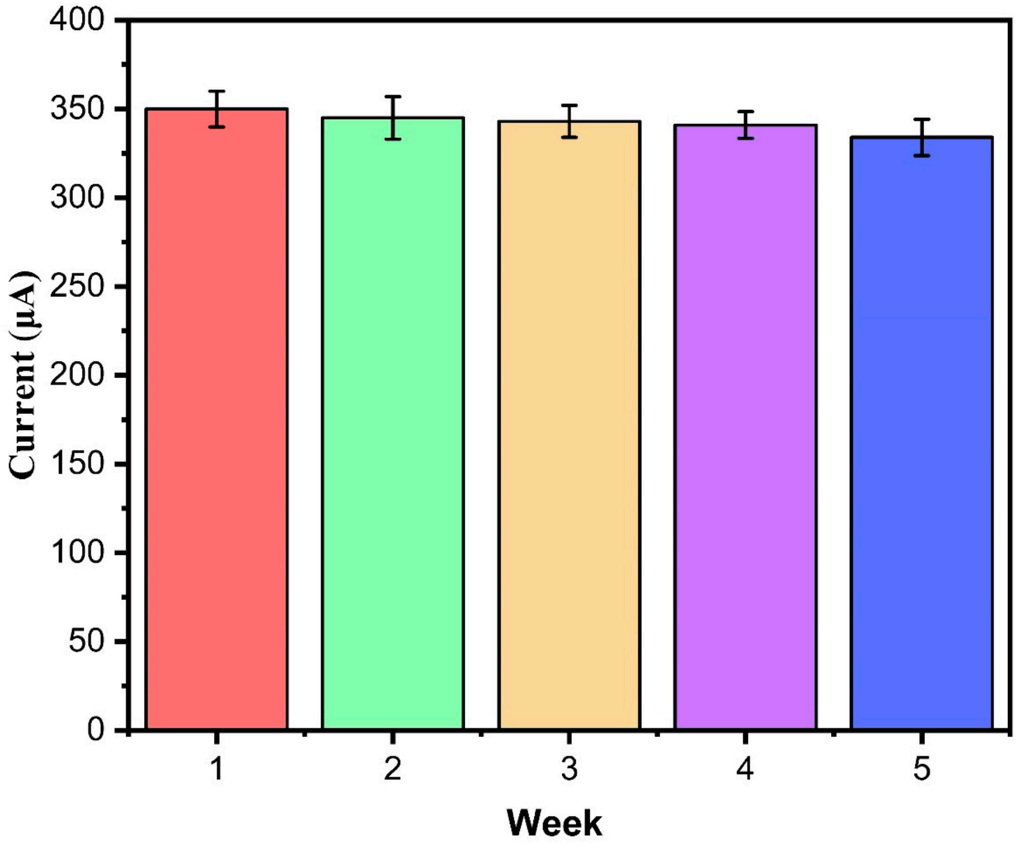

Figure S3. DPV signal measurement in the experimental setup in order to examine repeatability of GF electrodes.

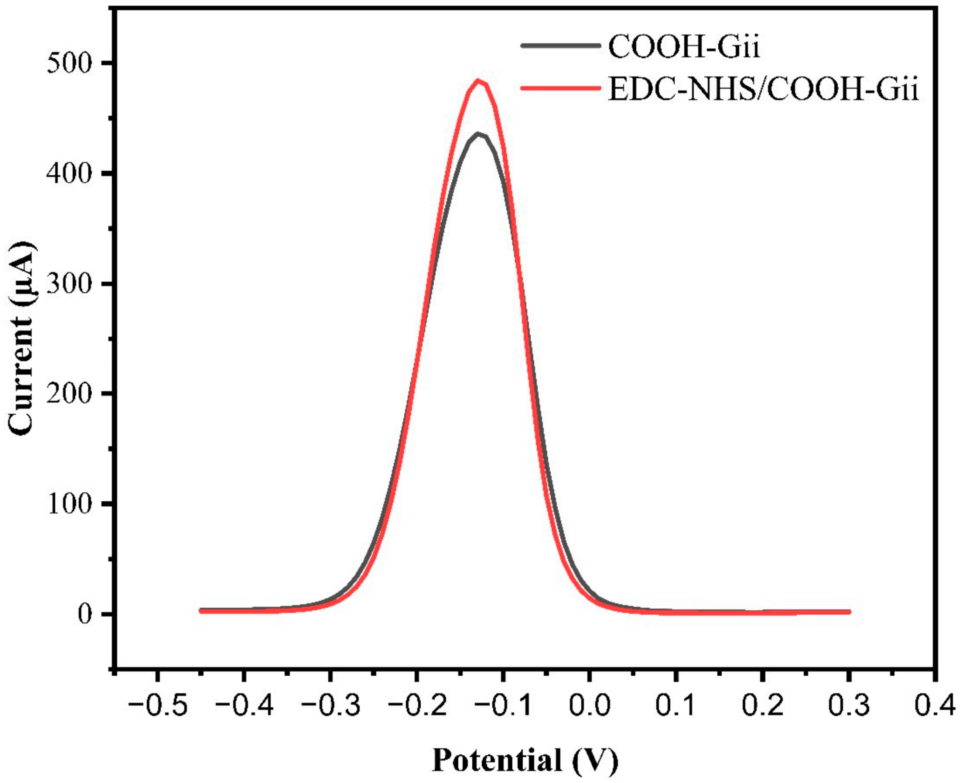

Figure S4. Compare the electrical characteristics of the electrode before and after the activation of the carboxyl group.

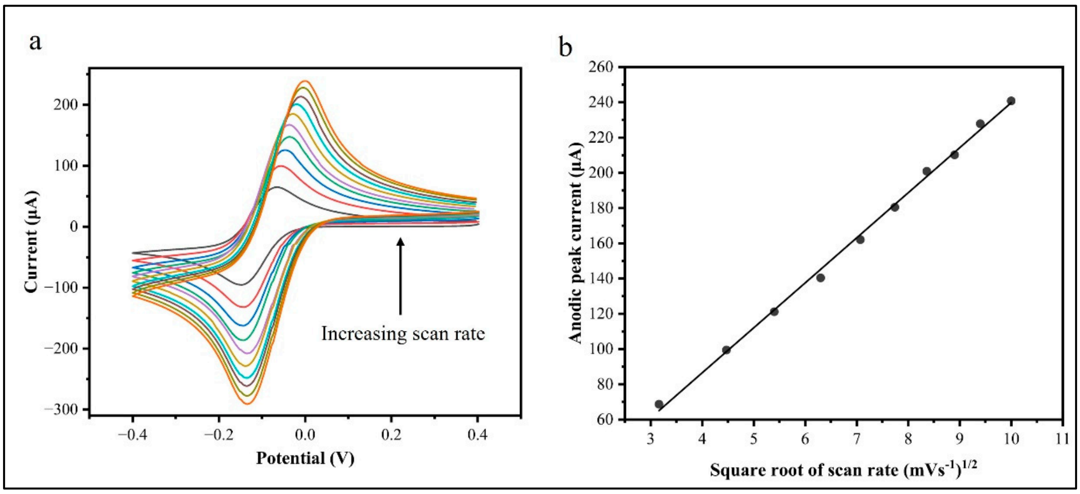

Figure S5. (a)Scan rate characterization from 0.01 V to 0.1 V by cyclic voltammetry characterization in a solution containing 10 mM $K_3[Fe(CN)_6]$ and 1 M KCl onto the BSA/mAb/EDC-NHS/COOH-GF biosensor. (b) linear curve of anodic peak current.

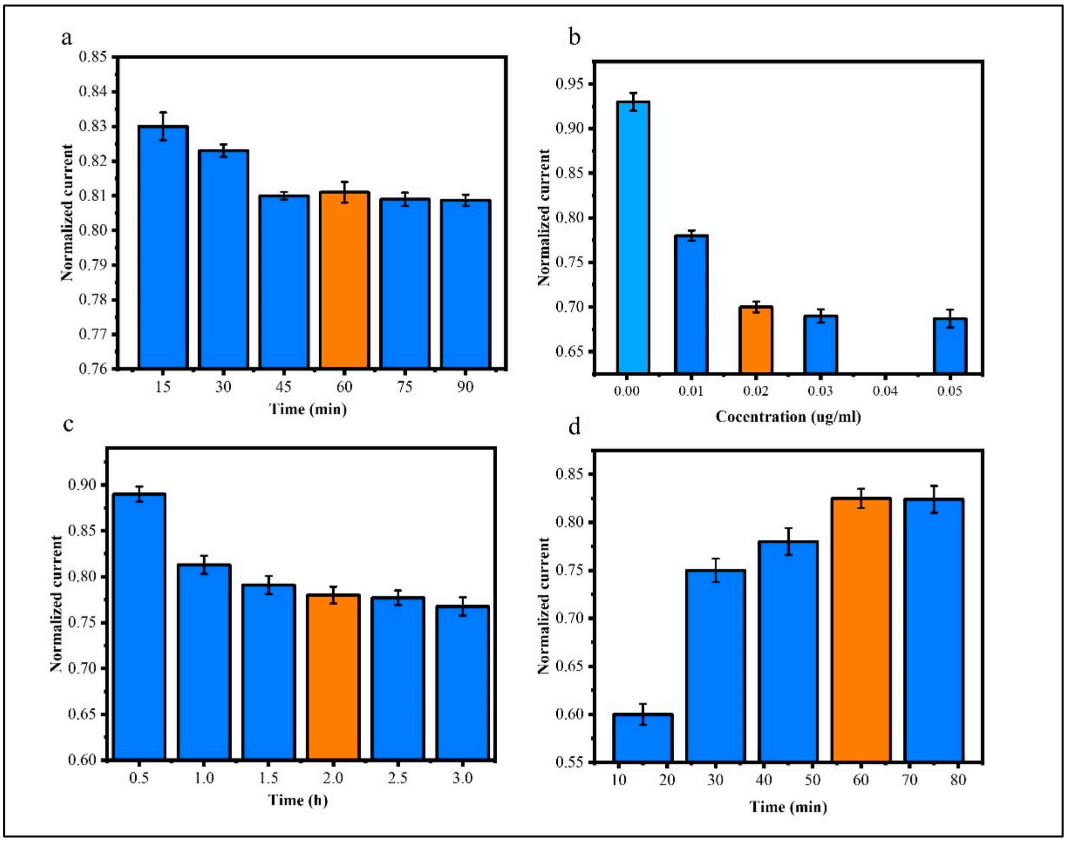

Figure S6. For the optimization of the experimental conditions, the DPV measurements were carried out in a solution containing 10 mM $K_3[Fe(CN)_6]$ and 1 M KCl using COOH-GF electrodes to investigate the effects of: (a) activation time of the COOH groups with the EDC-NHS cross-linker, (b) the concentration of tau-441 antibody, (c) the antibody immobilization time, (d) the antigen (tau-441 peptide) immobilization time
